# Preharvest Hydrogen Peroxide Treatment Delays Leaf Senescence of Chinese Flowering Cabbage During Storage by Reducing Water Loss and Activating Antioxidant Defense System

**DOI:** 10.3389/fpls.2022.856646

**Published:** 2022-03-31

**Authors:** Guang Wang, Miaomiao Peng, Yanjing Wang, Zhuosheng Chen, Shijiang Zhu

**Affiliations:** State Key Laboratory for Conservation and Utilization of Subtropical Agro-bioresources, Guangdong Provincial Key Laboratory of Potharvest Science of Fruits and Vegetables, Engineering Research Center of Southern Horticultural Products Preservation, Ministry of Education, College of Horticulture, South China Agricultural University, Guangzhou, China

**Keywords:** antioxidant defense system, Chinese flowering cabbage, hydrogen peroxide, leaf senescence, water loss

## Abstract

Leaf yellowing, an indicator of senescence, reduces commercial value of Chinese flowering cabbage after harvest. Hydrogen peroxide (H_2_O_2_) plays a dual role in mediating plant stress responses, but it is not clear whether and how it affects leaf senescence when exogenously stimulating the plants before harvest. Here, we found that preharvest application with low concentrations of H_2_O_2_ to root delays leaf senescence. Around 10 mM H_2_O_2_ reduced leaf yellowing rate by 8.2 and 26.4% relative to the control following 4 and 8 days storage, respectively. The H_2_O_2_-treated cabbages showed higher chlorophyll and lower relative expression of senescence-associated gene (SAG) *BrSAG12* than the control. Proteomic analysis revealed 118 and 204 differentially expressed proteins (DEPs) in H_2_O_2_-treated plants at 4 and 8 days of storage, respectively. The main DEPs are involved in chlorophyll degradation and synthesis, water deprivation, antioxidant activity, and protections on chloroplast membranes. Decline of water loss in H_2_O_2_-treated cabbages was coincide with increase of proline contents and modulation of leaf stomatal aperture. Alleviation of oxidative stress was indicated by suppression of respiratory burst oxidase homolog and upregulation of reactive oxygen species (ROS) scavenging-related genes. These results were also supported by the alleviation of lipid peroxidation and the protections on cell integrity and photochemical efficiency in H_2_O_2_-treated group. Collectively, preharvest H_2_O_2_ treatment alleviates water loss and activates antioxidant defense system, protects chloroplast membrane from oxidative damage, and ultimately delays leaf senescence during storage. This study provides novel insights into the roles of H_2_O_2_ for regulating leaf senescence of Chinese flowering cabbage.

## Introduction

Chinese flowering cabbage (*Brassica campestris* L. spp. *chinensis* var. *utilis* Tsen et Lee), a leafy vegetable with a flowering stem, is a specialty vegetable mainly produced in southern China and consumed worldwide (Zhang et al., 2013). However, the storage and transportation of Chinese flowering cabbage are restricted because the leaves are prone to yellowing after harvest. Leaf yellowing is a major indicator of leaf senescence. It is the main cause of a decline in its edibility and commodity value, leading to severe postharvest losses of the leafy vegetables (Porat et al., 2018). After harvest, vegetables are usually stored and transported under a weak light or even dark environment. Water loss and light deprivation are the key elements accelerating leaf senescence and strongly decrease shelf-life of vegetables (Liebsch and Keech, 2016). Chloroplast is the first organelle to be dismantled during leaf senescence. Degradation of chlorophyll is a signal involved in the senescence-associated degradation processes (Krieger-Liszkay et al., 2019). As an essential cofactor of the photosystem in chloroplasts, chlorophyll is important for photosynthesis. Research showed that the decline in photosynthetic activity and the downregulation of photosynthesis-associated genes expression are closely related to the increased expression of major senescence-associated genes (*SAGs*; Hensel et al., 1993). Therefore, the chlorophyll degradation and photosynthesis decline are important events during shade-induced leaf senescence.

Chlorophyll degradation in plant is basically promoted by a biochemical chlorophyll breakdown pathway, termed the “pheophorbide *a* oxygenase (PAO)/phyllobilin” pathway (Kuai et al., 2018). The pathway involves a series of chlorophyll catabolic enzymes (CCEs), including non-yellow coloring1 (NYC1), NYC1-like (NOL), stay-green (SGR), SGR-like (SGRL), pheophytin pheophorbide hydrolase (PPH), and pheide *a* oxidase (PAO; Kuai et al., 2018; Aubry et al., 2020). Chlorophyll breakdown process is regulated by internal and external stimuli during leaf senescence. Studies have confirmed that reactive oxygen species (ROS) production (oxidative burst) is closely related to aging metabolism in plants, and H_2_O_2_ over-accumulation is an important leaf senescence promoter (Guo et al., 2017). H_2_O_2_ plays a dual role in regulating plant physiological metabolism: It acts as a regulatory signal molecule in plant development and in abiotic or biotic stress responses at low concentrations (Rogers and Munné-Bosch, 2016). Exogenous H_2_O_2_ treatment can activate antioxidant defense systems and improve plant resistance to abiotic stress (Zhang et al., 2011). Strong antioxidant properties play an important role in delaying cell senescence. Postharvest treatments with H_2_O_2_ maintains fruit quality by activating the antioxidant defense system (Chumyam et al., 2019). However, to the best of the authors’ knowledge, there has been no research so far using exogenous H_2_O_2_ treatment preharvest for delaying leaf senescence in vegetables.

The aim of this study was to investigate the possible role of preharvest treatment with H_2_O_2_ on delaying leaf senescence in Chinese flowering cabbage. We studied the modulatory effects on leaf yellowing, lipid oxidation, cell integrity, chlorophyll content, photochemical efficiency and their relationships to oxidative stress and water loss. We also studied its regulation on proteins and expression levels of related key genes involved in ROS accumulation, chlorophyll synthesis/degradation, and photosynthesis capacity during cabbage storage. The findings of this study provide new insights into the mechanisms underlying exogenous H_2_O_2_-delayed leaf yellowing in Chinese flowering cabbage.

## Materials and Methods

### Preharvest Treatment of Chinese Flowering Cabbage With Hydrogen Peroxide

Seeds of Chinese flowering cabbage (Early-maturing cultivar: Youqingtian 405) were collected from the Guangdong Academy of Agricultural Sciences (Guangzhou, China). The seeds were seeded into pots using quartz sand as the growth substrate. Plant one seed in each pot (length 8.0 cm, width 8.0 cm, and depth 10.0 cm). The seedlings were regularly irrigated with modified Hoagland nutrient solution and cultivated under a 12-h light (1.5 × 10^4^ ± 200 lux)/12-h dark cycle at 25°C in an artificial climate room. At 35 days after sowing, the cabbages were randomly divided into two groups, the control and experiment groups. Root irrigation was performed with gradient concentrations of H_2_O_2_ (5, 10, 50, and 100 mM) in the experiment group. The control group was irrigated with pure water without H_2_O_2_. There were 150 cabbages in each group. The cabbages were harvested 24 h after treatment and then placed into plastic baskets that were lined with perforated polyethylene plastic bags (0.03 mm thickness). All cabbages were subsequently stored in an incubator at 15°C with a 12-h light (40 ± 10 lux)/12-h dark cycle. The harvested day was defined as 0 day, and they were observed and tested for related physiological parameters at days 0, 4, and 8 of storage. Three replications were performed for each treatment. Fresh leaves were frozen in liquid nitrogen and stored at −80°C for protein and RNA extraction.

### Postharvest Evaluation of Leaf Yellowing and Cell Integrity in Chinese Flowering Cabbage

The symptom of leaf yellowing was observed and photographed during storage. Leaf yellowing rate and the leaf yellowing index were used to estimate the degree of leaf senescence in Chinese flowering cabbage during storage (Zhang et al., 2010). Leaf yellowing rate of Chinese flowering cabbage is the proportion of yellowing leaves to the total number of leaves. The leaf yellowing index was assessed using a five-point scale as follows: 0, no yellowing; 1, yellowing covering <5% of the leaf surface; 2, yellowing covering 5–10% of the leaf surface; 3, yellowing covering 10–25% of the leaf surface; 4, yellowing covering 25–50% of the leaf surface; and 5, yellowing covering >50% of the leaf surface. Leaf yellowing index values were calculated based on these ratings using the following formula: leaf yellowing index = Σ(Nx × X)/(5 × ΣNx), where Nx is the total number of leaves observed in the rating X, and X = 0–5. Relative electrolytic leakage was used to estimate the cell integrity of Chinese flowering cabbage leaves. It was determined as reported previously (Liu et al., 2015).

### Evaluation of Chlorophyll Fluorescence Parameters and Chlorophyll Content

The photosystem II (PSII) maximum quantum yield (Fv/Fm) was measured using an imaging-PAM-M series chlorophyll fluorometer (Heinz Walz, Germany) equipped with a charge-coupled device camera to capture high-resolution digital images of the fluorescence (Khan et al., 2019). The PSII maximum quantum efficiency was calculated using the following formula: Fv/Fm = (Fm-Fo)/Fm.

To evaluate the chlorophyll (*a, b*) content, 2.0 g (fresh weight) of leaf tissue was ground to a fine powder with liquid nitrogen, and the chlorophyll was extracted with 80% cold acetone overnight at 4°C (Nyoki and Ndakidemi, 2014). The extract was spectrophotometrically quantified by measuring its optical density at 663 and 645 nm with a UV-2450 spectrophotometer (Shimadzu, Japan).

### Measurement of Stomatal Aperture and Water Loss in Chinese Flowering Cabbage

To determine the regulatory effect of H_2_O_2_ on the apertures, we measured the stomatal apertures at days 4 and 8 of storage using a colorless imprint method (Wu and Zhao, 2017). The photographs were imaged with a bright-field microscopy. The average aperture widths of >100 stomata in each treatment were quantified. Water loss was calculated by subtracting the sample weights at days 4 and 8 of storage from those recorded at day 0, and presented as a percentage of the initial weight. The fresh weight of Chinese flowering cabbage samples during postharvest storage were measured using a 0.01 g accuracy scale.

### Detection of O_2_^−^ and H_2_O_2_ Content and Location in Leaves During Storage

For histochemical detection of the localization of O_2_^−^ and H_2_O_2_ in leaves during storage, we used nitro blue tetrazolium (NBT) and 3,3′-diaminobenzidine (DAB) staining methods according to our previous study (Wang et al., 2018). A spectrophotometric method was used to analyze the contents of O_2_^−^ and H_2_O_2_ in leaves during storage (Yu et al., 2019).

### Measurement of Malondialdehyde Contents During Storage

Malondialdehyde (MDA) content was assayed using a kit produced by Nanjing Jiancheng Institute of Biotechnology (Jiangsu, China). Briefly, the leaves of cabbages were ground to powder with liquid nitrogen. Then, 1.0 g of powder was dissolved in 10 ml of 5% trichloroacetic acid, and then centrifuged at 5,000 × *g* at 4°C for 10 min. The supernatants were collected in new tubes and mixed with 0.67% thiobarbituric acid. The mixtures were boiled in water for 30 min and then centrifuged at 10,000 × *g* for 10 min. Absorbance of the supernatants was measured at 532 and 600 nm.

### RNA Isolation and Quantitative Real-Time PCR

Total RNA was extracted from leaves using the RNAprep Pure Plant Kit (TianGen, China). cDNA was produced from RNA using the 1st Strand cDNA Synthesis SuperMix (Yeasen, Shanghai, China). The gene-specific primers were designed with the Primer-BLAST. All primers used in this study are listed in Supplementary Table 1. Quantitative real-time PCR (qRT-PCR) was performed using a Bio-Rad CFX96 Real-Time PCR System. Reactions were carried out using the SYBR® Green Master Mix (Yeasen, Shanghai, China). Data were normalized using the cycle threshold (Ct) value corresponding to the reference gene (Tan et al., 2019).

### Protein Extraction and Trypsin Digestion

The Chinese flowering cabbage leaves of 10 mM H_2_O_2_ treatment and control groups were sampled at day 4 and 8 of storage. Leaves were ground to powder with liquid nitrogen and added to four times volume of lysis buffer (including 3 μM TSA, 10 mM dithiothreitol, 50 mM NAM, and 1.0% protease inhibitor). After being sonicated on ice for 15 min, the mixture was mixed with an equal volume of phenol (stabilized with 0.1 M tris buffer) and centrifuged at 5,500 × *g* for 12 min at 4°C. The proteins in supernatant were precipitated with cold ammonium acetate/methanol and then collected by centrifugation at 12,000 × *g* for 15 min at 4°C. For digestion, the protein sample was redissolved with 200 mM triethylammonium bicarbonate, followed by sonication and the addition of trypsin in a 1:50 trypsin-to-protein mass ratio. After digestion overnight, the protein solution was reduced with dithiothreitol and then alkylated with iodoacetamide in the dark.

### Liquid Chromatography–Tandem Mass Spectrometry Quantitative Proteomic Analysis

For liquid chromatography–tandem mass spectrometry (LC–MS/MS) analyses, the tryptic peptides were dissolved in 0.1% formic acid. The peptide fragments were separated using a Bruker nanoElute ultra-performance liquid chromatography system (UPLC). Using solvent A (containing 0.1% formic acid and 2.0% acetonitrile) and solvent B (containing 0.1% formic acid and 100% acetonitrile) as the mobile phase, the LC linear gradient elution was established as follows: 0–70 min, 6–24% B; 70–84 min, 24–35% B; 84–87 min, 35–80% B; 87–90 min, 80% B; at a constant flow rate of 450 nl/min. After separation with UPLC, the peptides were subjected to the capillary ion source for ionization and then analyzed using a timsTOF Pro (Bruker Daltonics, MA, United States) mass spectrometer. The electrospray was applied at 2.0 kV, with an MS/MS scan range of 100 to 1700 m/z.

### Database Search and Bioinformatic Analysis

The proteomic database was searched, and the MS/MS data were analyzed using the Maxquant search engine (v.1.6.6.0). The mass tolerance for precursor ions was set at 20 parts per million (ppm) in both the first and main searches. Oxidation on methionine and acetylation on the protein N-terminal were specified as the variable modifications, and carbamidomethyl on cysteine was specified as a fixed modification. The differentially regulated proteins were quantitated using the label-free quantitative (LFQ) method, and the false discovery rates of protein and peptide spectrum match identification were adjusted to 1.0%.

A ratio of fold change >1.5 was set to identify differentially regulated proteins with a *t*-test value of *p* < 0.05. The Gene Ontology (GO) annotation proteome was derived from the UniProt-GOA database. The Kyoto Encyclopedia of Genes and Genomes (KEGG) database was used to identify enriched pathways and to determine the enrichment of the differentially regulated proteins against all identified proteins; the pathways with a corrected value of *p* < 0.05 were considered significant. The annotation results were mapped to the KEGG pathway database using the KEGG mapper. The subcellular localization of the submitted proteins was annotated using WoLF PSORT software to predict subcellular localization (Horton et al., 2007).

### Statistical Analysis

All experiments were performed in triplicate. The proteomic analysis was performed using a timsTOF Pro instrument with three technical replicates. Results are presented as means ± SD. The parameter means were analyzed with Duncan’s multiple range test using the SPSS 19.0 software (IBM Corp., Armonk, NY, United States). The differences between the means at *p* < 0.05 were considered statistically significant.

## Results

### Preharvest Application With Exogenous H_2_O_2_ Alleviates Leaf Yellowing and Protects the Cell Integrity of Chinese Flowering Cabbage

Leaf yellowing was evaluated by calculating leaf yellowing rate and leaf yellowing index. Leaf yellowing rate reflects the ratio of yellowing leaves to total leaves, and the leaf yellowing index is regarded as an indicator of the degree of leaf yellowing. Leaf yellowing in harvested Chinese flowering cabbage was affected by different concentrations of H_2_O_2_ applied to roots before harvest. Hydrogen peroxide plays a dual role in regulating leaf senescence in Chinese flowering cabbage. It was showed that preharvest treatment with 100 mM H_2_O_2_ markedly accelerated leaf yellowing, while low concentrations of H_2_O_2_ (<50 mM) alleviated leaf yellowing at day 8 of storage. Treatment of 10 mM H_2_O_2_ showed the best effects on reducing leaf yellowing rate and leaf yellowing index (Supplementary Figure S.1). Therefore, 10 mM H_2_O_2_ treatment was selected for the following study.

**Figure 1:**
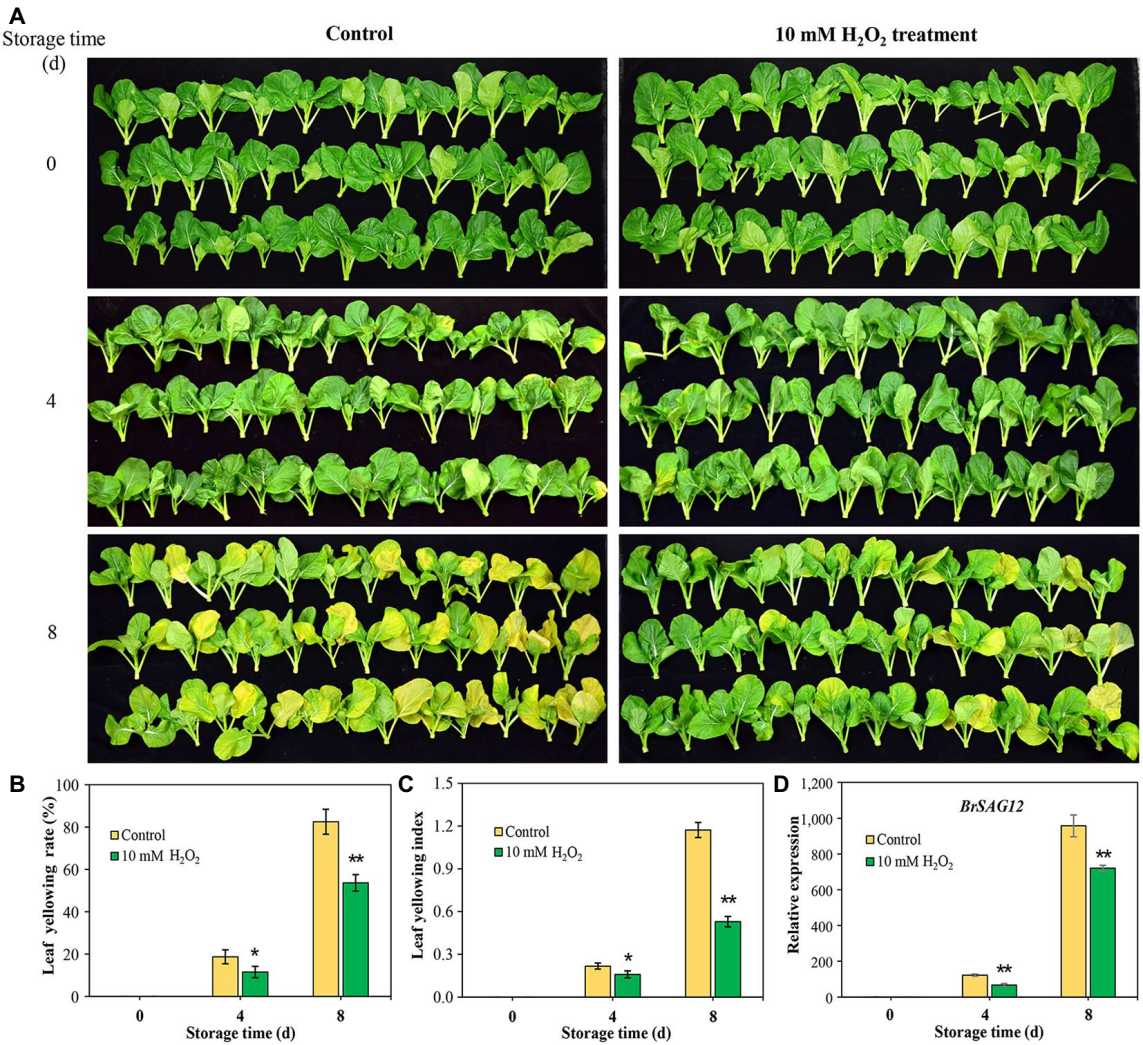
Exogenous hydrogen peroxide (10 mM H_2_O_2_) alleviates leaf senescence in Chinese flowering cabbage, as is shown by leaf yellowing symptom **(A)**, leaf yellowing rate **(B)**, leaf yellowing index **(C)**, and relative expression of senescence-associated gene (SAG) *BrSAG12* **(D)** in leaves during storage. Error bars represent one SD. Asterisks indicate that the mean values of H_2_O_2_ treatment are significantly different from those of the control (^*^p < 0.05, ^**^p < 0.01), according to Duncan’s multiple range test.

Images of Chinese flowering cabbage showed that the control samples exhibited visible yellowing symptom with time during storage (Figure 1A), and the yellowing rates were 19.7 and 82.5% at days 4 and 8, respectively (Figure 1B). However, preharvest application with 10 mM H_2_O_2_ to root alleviated leaf yellowing, with the leaf yellowing rate being reduced by 8.2 and 26.4% at the two time points monitored (Figure 1B). Moreover, leaf yellowing indexes in H_2_O_2_ group were markedly low throughout the storage period compared to that in the control sample (Figure 1C). The senescence marker gene *BrSAG12* is considered a reference for evaluating leaf senescence (Carrión et al., 2013). The results here showed that the expression levels of *BrSAG12* in H_2_O_2_-treated cabbages were markedly lower than that of the control during storage, indicating leaf senescence was delayed by preharvest H_2_O_2_ treatment (Figure 1D).

### Sub-localization and GO Functional Annotations of H_2_O_2_-Induced Differentially Regulated Proteins

**Figure 2:**
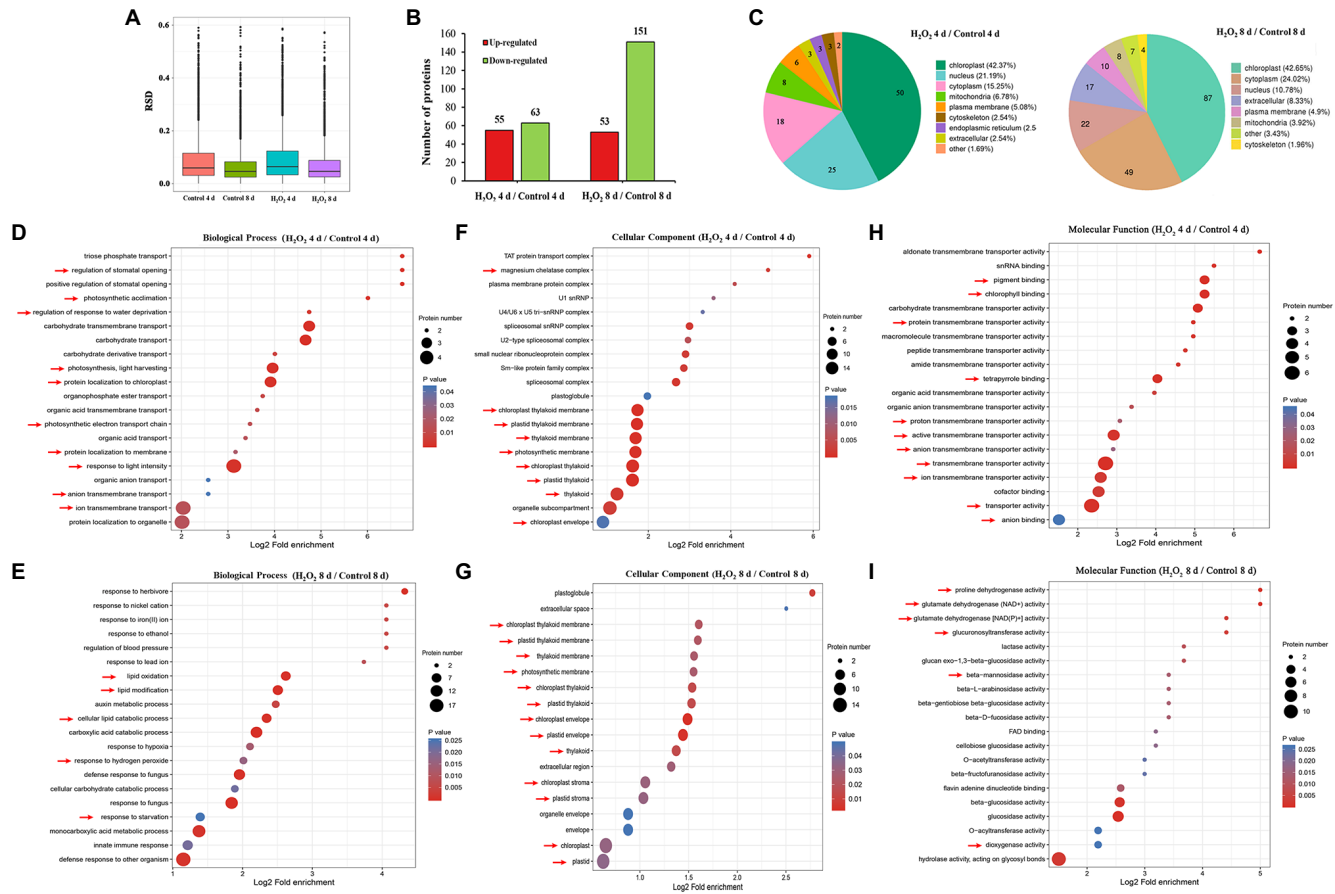
Proteomic analysis (10 mM H_2_O_2_/Control) of Chinese flowering cabbage leaves during storage. The relative SD (RSD) between repeated samples **(A)**. The differentially regulated proteins of Chinese flowering cabbage leaf in the H_2_O_2_ treatment group, a 1.5-fold change (*p* < 0.05) compared to controls **(B)**. The sub-cellularly localization of H_2_O_2_-induced differentially regulated proteins **(C)**. The biological process **(D,E)**, cellular components **(F,G)**, and molecular function **(H,I)** functional annotations of total differentially expressed proteins of Chinese flowering cabbage leaf at day 4 and day 8 of storage.

To analyze the effects of preharvest H_2_O_2_ treatment in protein levels during leaf senescence, the differentially expressed proteins (DEPs) in leaves during storage were detected with proteomics approach. In comparison with the control plants, 118 and 204 DEPs were identified in 10 mM H_2_O_2_ treatment group at 4 and 8 days, respectively (Figures 2A,B), of which, 42.37 and 42.65% were localized sub-cellularly in the chloroplasts (Figure 2C). These results suggested that the chloroplasts are the major site in leaves influenced by preharvest application of exogenous H_2_O_2_.

Functional category analysis of the total DEPs in leaves was conducted based on GO functional annotations (including biological processes, cellular components, and molecular functions). Biological process annotation revealed that the main DEPs at day 4 during storage were involved in response to stimulations (Supplementary Figure S.2), including response to water deprivation, regulation of stomatal opening, photosynthetic acclimation, photosynthesis, light harvesting, and protein localization to chloroplast (Figure 2D). The main DEPs at day 8 of storage were involved in response to oxidative stress, including lipid oxidation, lipid modification, response to hydrogen peroxide, and response to starvation (Figure 2E). Cellular components annotation revealed that 64 of the DEPs at day 4 and 47 of the DEPs at day 8 were involved in the components of magnesium chelatase, chloroplast thylakoid membrane, plastid thylakoid membrane, thylakoid membrane, photosynthetic membrane, chloroplast thylakoid, plastid thylakoid, organelle subcompartment, and chloroplast envelope (Figures 2F,G). Molecular functions annotation showed that most of the DEPs in were involved in catalytic activity, transporter activity, antioxidant activity, and molecular function regulator (Supplementary Figure S.2; Figures 2H,I). These results imply that H_2_O_2_ treatment may be involved in regulating water loss, activating antioxidant defense system, and contributing to the stability of chloroplast membrane system.

### Preharvest H_2_O_2_ Treatment Protects Light-Harvesting Proteins in Leaves

**Figure 3:**
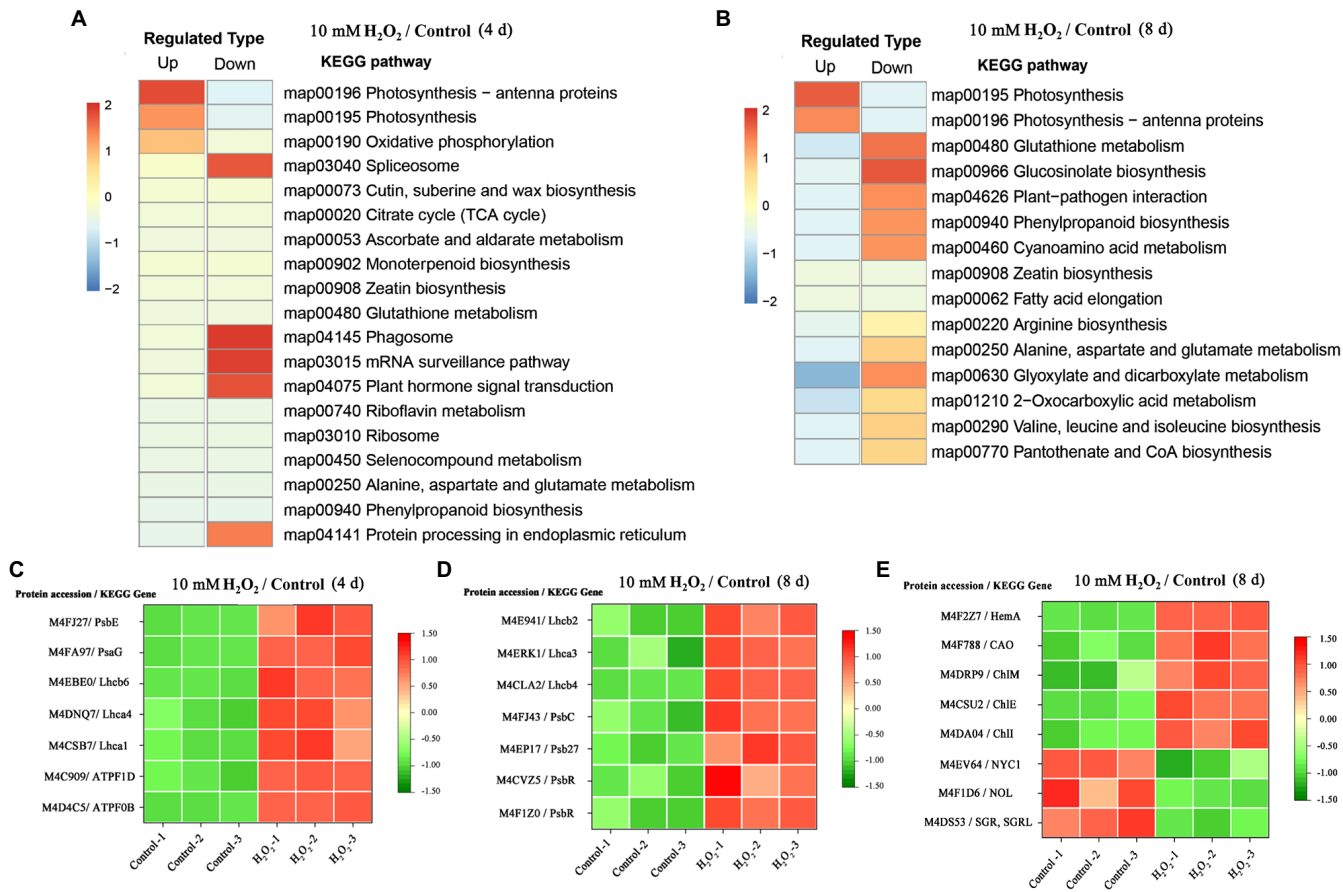
The regulation of H_2_O_2_ treatment in chlorophyll synthesis and degradation process. Enrichment of Kyoto Encyclopedia of Genes and Genomes (KEGG) pathways of upregulated proteins and downregulated proteins of Chinese flowering cabbage leaf at day 4 **(A)** and day 8 of storage **(B)**. Heat map of differentially regulated proteins involving in photosynthesis and light-harvesting during storage **(C,D)**; and chlorophyll synthesis and degradation **(E)** at day 8 of storage.

Enrichment of KEGG pathways analysis showed that, at both day 4 and day 8 of storage of Chinese flowering cabbage, the upregulated DEPs were involved in light harvesting and photosynthesis-related pathway (Figures 3A,B). The light-harvesting complex (Lhc) proteins, including Lhcb6, Lhca1, Lhca4, Lhcb2, Lhcb4, and Lhca3 (Figures 3C,D), and photosynthesis-related proteins, including photosystem I subunit V (PsaG), photosystem II cytochrome b559 subunit alpha (PsbE), PS II 10 kDa protein (PsbR), PS II Psb27 protein (Psb27), and PS II CP43 chlorophyll apoprotein (PsbC) were enhanced in H_2_O_2_-treated plants in comparison with the control. In addition, F-type H + -transporting ATPase subunit b (ATPF0B) and F-type H + -transporting ATPase subunit delta (ATPF1D) were upregulated in the H_2_O_2_-treated plants at day 4 of storage (Figure 3C).

### Preharvest H_2_O_2_ Treatment Preserves Chlorophyll Synthesis and Slows Down the Chlorophyll Degradation Process

Proteomic analysis showed that glutamyl-tRNA reductase (HemA), magnesium ion chelating enzyme (ChlI), chlorophyllide *a* oxygenase (CAO), magnesium-protoporphyrin IX monomethyl ester (oxidative) cyclase (ChlE), and magnesium-protoporphyrin O-methyltransferase (ChlM) were upregulated by H_2_O_2_ treatment (Figure 3E). These enzymes are involved in chlorophyll biosynthesis pathway; among them, HemA, ChlI, and CAO are the key rate-limiting enzymes, suggesting that preharvest application of H_2_O_2_ may modulate chlorophyll synthesis during storage.

At day 8 in storage, chlorophyll(ide) *b* reductase (NYC1 or NOL) and magnesium-free chelatase (SGR or SGRL) were suppressed by preharvest treatment with H_2_O_2_ (Figure 3E). These proteins are correlated with chlorophyll degradation, suggesting that chlorophyll degradation in leaves was suppressed during storage. This was verified by results of qRT-PCR, which showed that the expression of chlorophyll catabolic genes (*BrNYC1*, *BrSGR1*, *BrSGR2*, *BrSGRL*, and *BrPPH*) were all downregulated in the H_2_O_2_-treated plants in comparison with the control at days 4 and 8 after harvest (Supplementary Figure S.3).

### Preharvest H_2_O_2_ Treatment Preserves Chlorophyll Contents and Photochemical Efficiency of Leaves

**Figure 4:**
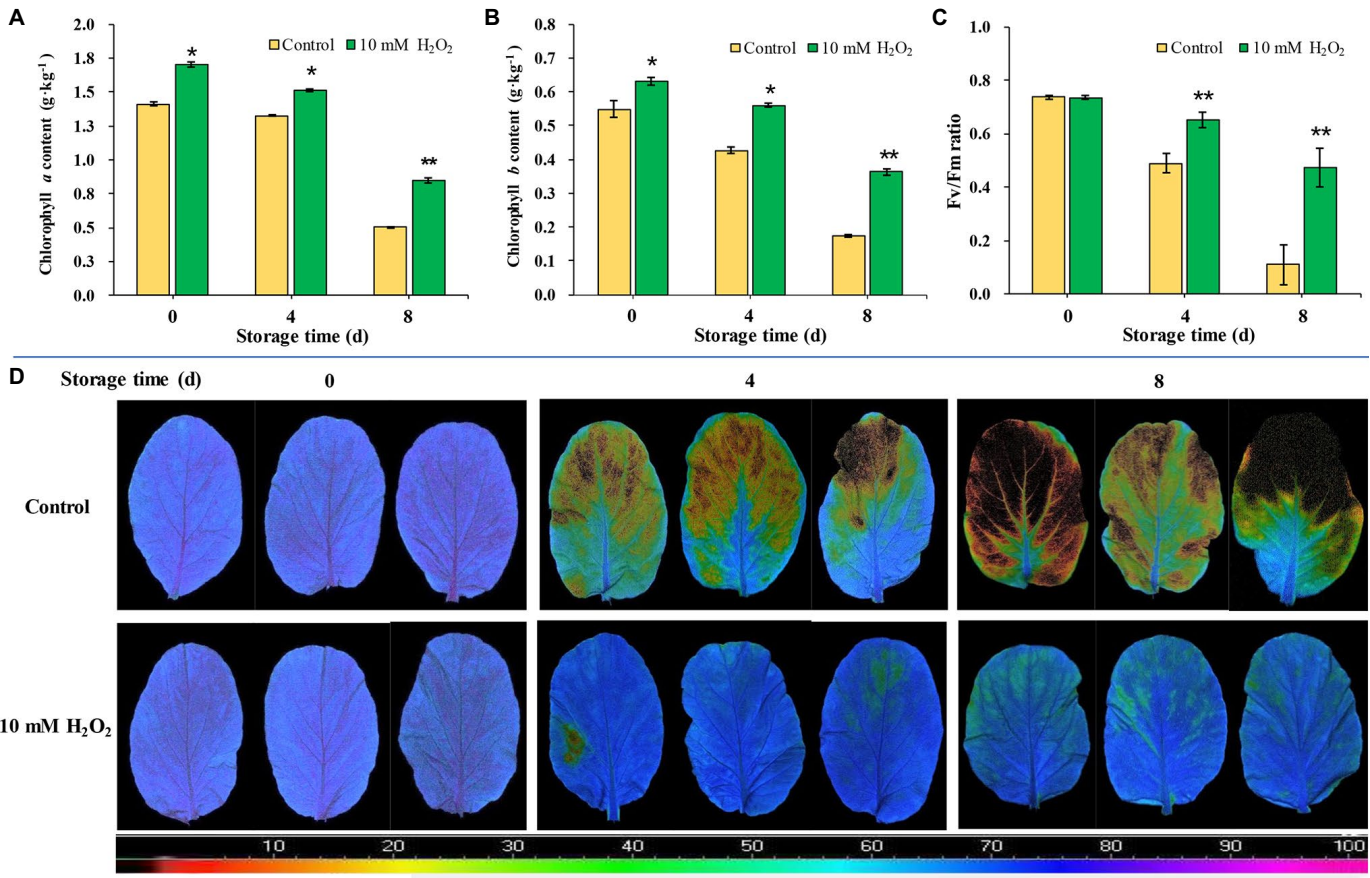
The effects of preharvest application of 10 mM H_2_O_2_ on Chlorophyll contents and photochemical efficiency of Chinese flowering cabbage leaves during storage. The chlorophyll *a* **(A)** and chlorophyll *b* **(B)** contents, Fv/Fm value **(C)**, and chlorophyll fluorescence images **(D)** of Chinese flowering cabbage leaves during storage. The images were color-coded based on the Fv/Fm value. The blue- to purple-coded area indicates healthy leaves, whereas the yellow to the dark-red-coded area indicates disfunction of photosystem II function in leaves. Error bars represent one SD. Asterisks indicate that the mean values of H_2_O_2_ treatment are significantly different from those of the control (^*^p < 0.05, ^**^p < 0.01), according to Duncan’s multiple range test.

Chlorophyll contents in leaves markedly decrease as leaf got yellowed during storage. The chlorophyll contents of leaves during storage were consistent with their phenotype. However, H_2_O_2_-treated plants showed higher contents of chlorophylls *a* and *b* than the control (Figures 4A,B). We monitored temporal changes in leaf photosynthetic parameters using non-invasive chlorophyll fluorescence images. Fv/Fm is one of the most widely used chlorophyll fluorescence parameters highly correlated with the maximum photochemical efficiency of chloroplasts (Fang et al., 2020). The results showed that Fv/Fm value decreased rapidly during aging of leaves; Fv/Fm value in control group decreased from 0.74 at day 0 to 0.49 at day 4 and further decreased to 0.11 at day 8 after harvest. However, the Fv/Fm value in the H_2_O_2_ treatment group stayed between 0.74 and 0.51 during the same period monitored (Figures 4C,D). These results indicate that the chloroplast function in H_2_O_2_ treatment group was preserved during storage. The changes of Fv/Fm value were consistent with leaf yellowing symptom, and relative expression of senescence-associated gene *BrSAG12* in leaves during storage (Figures 1A–D). These results are closely related to the effects on chlorophyll content and the synthesis/degradation metabolism (Figure 3; Supplementary Figure S.3).

### Hydrogen Peroxide Modulates Leaf Stomatal Aperture and Reduces Water Loss

**Figure 5:**
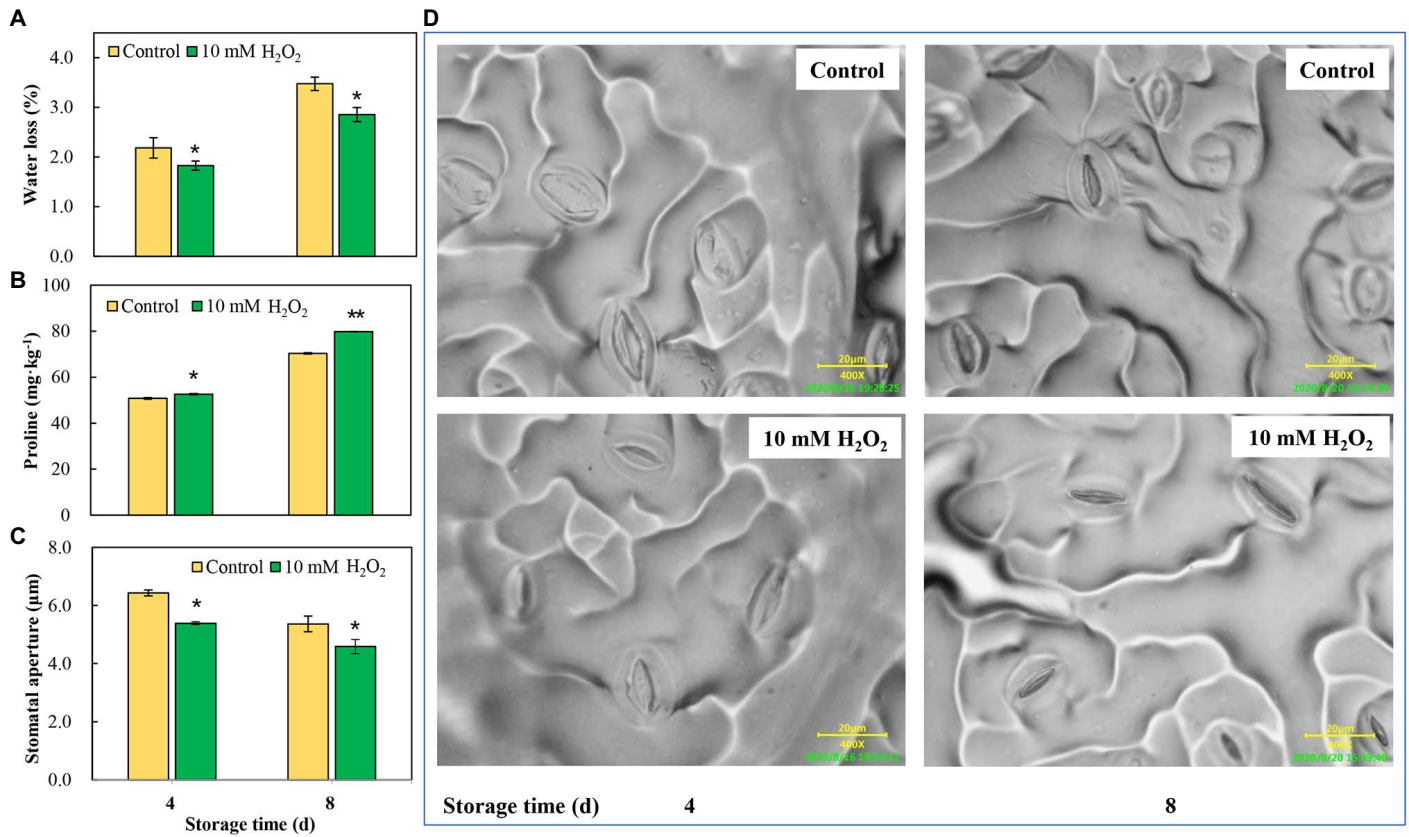
The effects of preharvest application of 10 mM H_2_O_2_ in water retention of Chinese flowering cabbage leaves during storage. The regulation of water loss **(A)**, proline content **(B)** and stomatal apertures **(C,D)** of Chinese flowering cabbage leaves during storage. Error bars represent one SD. Asterisks indicate that the mean values of H_2_O_2_ treatment are significantly different from those of the control (^*^p < 0.05, ^**^p < 0.01), according to Duncan’s multiple range test.

Chinese flowering cabbage is particularly prone to water loss during storage due to its high leaf surface area and the increased water stress that occurs in the leaves. Our results showed that water loss increased with time during storage in both the control and treatment groups. However, water loss was reduced by preharvest treatment with H_2_O_2_ (Figure 5A). Interestingly, we found that proline concentrations in leaves were negatively correlated with their water loss during storage (Figure 5B). Moreover, the water loss in the treatments was in line with the stomatal aperture, and H_2_O_2_ treatment reduced the stomatal aperture at days 4 and 8 after harvest (Figures 5C,D). These results can be accounted for by the proteomic analysis results which showed that main DEPs induced by H_2_O_2_ treatment were involved in regulation of stomatal opening, photosynthetic acclimation, and response to water deprivation (Figure 2D). The modulation of water loss during storage was correlated with leaf senescence process (Figure 1D). The effects of H_2_O_2_ treatment on water retention is also closely related to the changes in chlorophyll content and maximum photochemical efficiency during storage (Figure 4).

### Preharvest H_2_O_2_ Treatment Reduces the Oxidative Stress in Leaf Tissues

**Figure 6:**
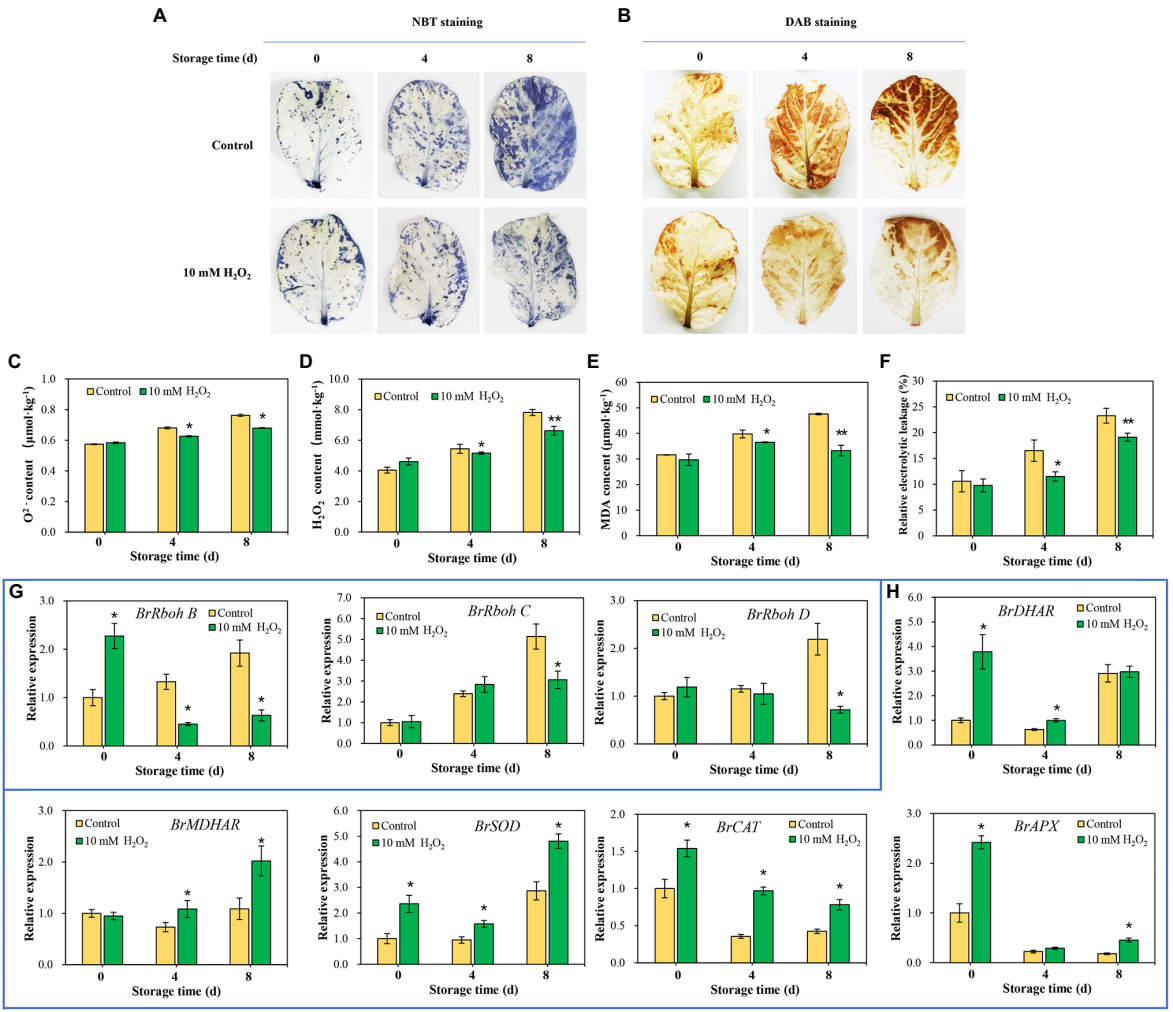
Effects of preharvest H_2_O_2_ treatment on reactive oxygen species (ROS) accumulation and antioxidant capacity of leaves during storage. Location of O_2_ **(A)** and H_2_O_2_ **(B)** in leaves were detected with nitro blue tetrazolium (NBT) and 3,3′-diaminobenzidine (DAB), respectively. Changes in the contents of O_2_^−^ **(C)**, H_2_O_2_ **(D)**, malondialdehyde (MDA; **E**), and relative electrolytic leakage **(F)** at days 0, 4 and 8 after harvest. The relative expression levels of ROS biosynthesis genes, including *BrRbohB*, *BrRbohC*, and *BrRbohD* **(G)**, and ROS scavenging-associated genes, including *BrDHAR*, *BrMDHAR*, *BrSOD*, *BrCAT*, and *BrAPX* **(H)** during storage. Error bars represent one SD. Asterisks indicate that the mean values of H_2_O_2_ treatment are significantly different from those of the control (^*^p < 0.05, ^**^p < 0.01), according to Duncan’s multiple range test.

Endogenous ROS is produced by respiratory burst oxidase homologs (Rbohs) and act as a senescence accelerator in plant tissues. The oxidative damage induced by ROS in tissues is an important index for evaluating the process of leaf senescing (Niu et al., 2020). Here, we found that the superoxide anion (O_2_^−^) and hydrogen peroxide (H_2_O_2_) were accumulated, accompanied by an increase in MDA in the tissues, with leaf aging during storage. However, preharvest H_2_O_2_ treatment inhibited the increase in O_2_^−^ and H_2_O_2_ (Figures 6A–D), as well as the accumulation of MDA (Figure 6E). Relative electrolytic leakage was measured to investigate cell integrity. The results showed that the changes of relative electrolytic leakage are correlated with the MDA accumulation in leaves. Relative electrolytic leakage in the control group was higher than that in the H_2_O_2_ treatment group (Figure 6F), indicating severe membrane oxidative damage in the control group. Conversely, the cell integrity was protected in the H_2_O_2_ treatment group, suggesting that preharvest treatment with H_2_O_2_ reduces cell oxidative damage during storage. The attenuation of oxidative stress may be attributed to the activation of antioxidant system by low H_2_O_2_ treatment. It involves in the upregulation of ROS scavenging-related genes (*BrDHAR*, *BrMDHAR*, *BrSOD*, *BrCAT*, and *BrAPX*) and suppression of ROS biosynthesis-related genes (*BrRbohB*, *BrRbohC*, and *BrRbohD*; Figures 6G,H). These regulations suppressed the accumulation of ROS in tissues, leading to a decrease of MDA accumulation and delay of leaf senescence.

## Discussion

Leaf senescence is the primary factor affecting commodity value of leafy vegetables during postharvest storage and distribution. Over the past decades, researchers have been attempting to develop techniques to delay leaf senescence. H_2_O_2_ is an important signaling molecule playing a dual role in regulating plant senescence. Excessive accumulation of H_2_O_2_ can accelerate leaf senescence (Guo et al., 2017). However, application with low concentration of exogenous H_2_O_2_ can activate the antioxidant enzymes under environmental stresses (Zhang et al., 2011). The results here showed that preharvest application of 10 mM H_2_O_2_ to root effectively alleviated leaf yellowing and prolonged the shelf life of Chinese flowering cabbage after harvest. The retardation of leaf senescence during storage was manifested in lower oxidative stress, water loss, and leaf yellowing indexes, as well as higher chlorophyll levels and photosynthetic capacity. To the best of our knowledge, this is the first time showing that treating vegetable roots with low H_2_O_2_ before harvest can delay leaf senescence during storage.

Leaf senescence, regarded as the final stage of leaf development, is a highly controlled process requiring massive transcriptional regulations (Liebsch and Keech, 2016). The upregulated genes involves in the senescence process have been designated as *SAGs*, among which *SAG12* is a senescence-specific marker gene and be widely used as the reference for evaluating the leaf senescence (Carrión et al., 2013). The expression of *BrSAG12* in Chinese flowering cabbage is markedly upregulated as leaf got yellowed during storage. However, the upregulation of *BrSAG12* was notably suppressed in H_2_O_2_ treatment group in comparison to control group (Figures 1A–D). Leaf yellowing, in considered a symptom of senescence, involves disassembly of chloroplast and breakdown of related protein complexes (Brouwer et al., 2012). Chloroplast is the first organelle to be dismantled during leaf senescence, and chlorophyll degradation is one of the most important events (Schippers et al., 2015). In the present study, the contents of chlorophyll *a* and *b* in leaves during storage declined with the upregulating of *BrSAG12*. However, both the chlorophyll *a* and *b* were preserved in the H_2_O_2_-treated plants (Figures 4A,B). These results suggest that preharvest treatment with H_2_O_2_ delay leaf senescence and alleviate leaf yellowing during storage.

**Figure 7:**
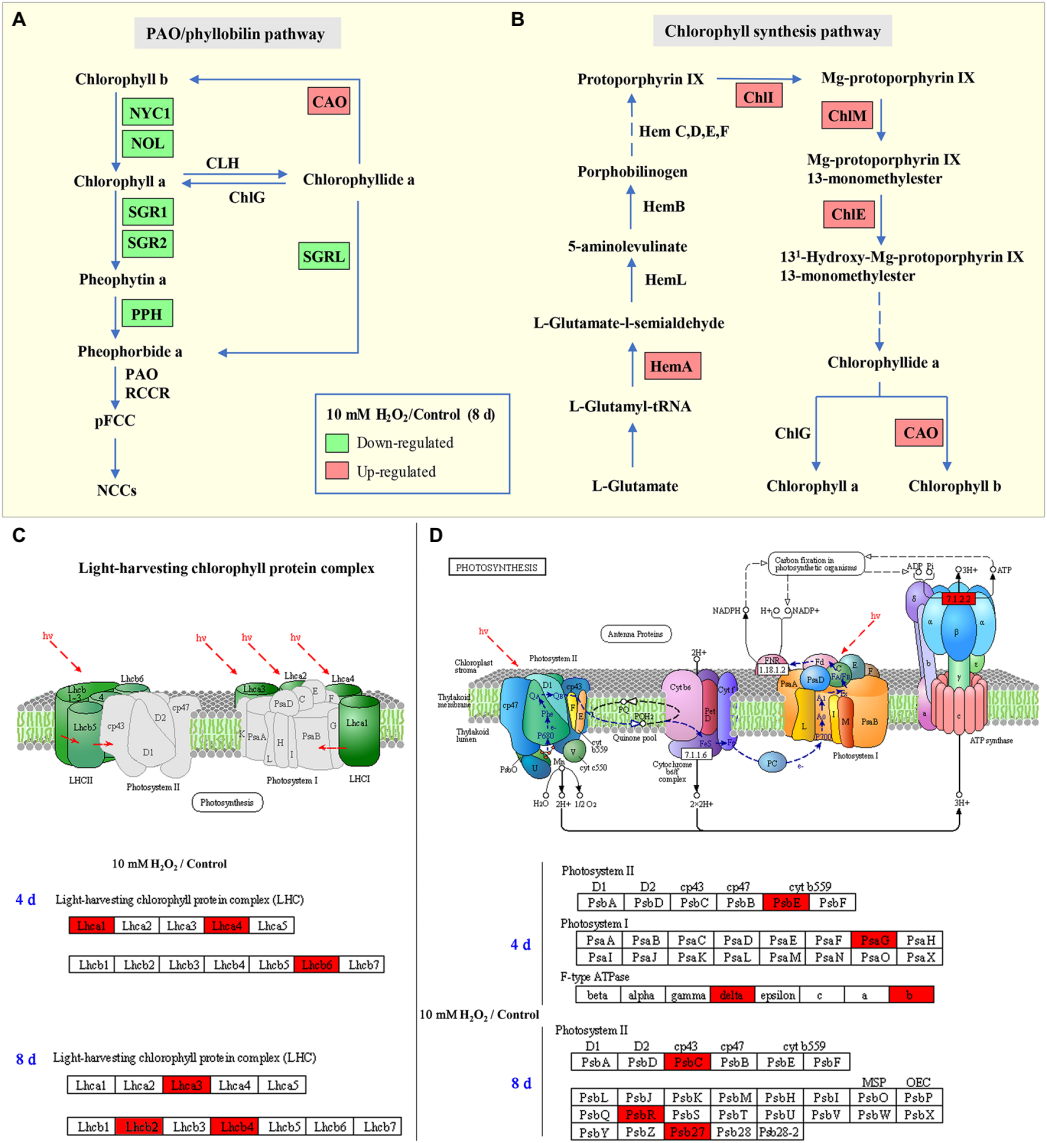
Schematic map of chlorophyll degradation **(A)** and biosynthesis **(B)** pathways, light-harvesting complex **(C)**, and photosynthesis **(D)** in Chinese flowering cabbage leaves. Red color indicates upregulation and green color indicates downregulation of proteins/genes in the H_2_O_2_ treatment group relative to the control group. Lhc: light-harvesting complex; HemA: Glutamyl-tRNA reductase; ChlI: magnesium ion chelating enzyme; ChlM: magnesium-protoporphyrin O-methyltransferase; ChlE: magnesium-protoporphyrin IX monomethyl ester cyclase; ChlG: chlorophyll/bacteriochlorophyll *a* synthase; CAO: chlorophyllide *a* oxygenase; NYC1: non-yellow coloring 1; NOL: NYC1-like; SGR: stay-green; SGRL: SGR-like; and PPH: pheophytin pheophorbide hydrolase.

During leaf senescence, the chlorophyll generally decline as the chlorophyll catabolic genes are preferentially enhanced and chlorophyll biosynthesis genes are down-regulated (Wang et al., 2020). The “PAO/phyllobilin pathway” is a dedicated pathway responsible for the degradation of chlorophyll (Figure 7A), which is associated with SGRs and some CCEs, including NOL, NYC, and PAO (Kuai et al., 2018). Studies have demonstrated that *SGRs* or *NYC* mutants showed a stay-green phenotype in *Arabidopsis*, while the over-expression of *SGRs* induces premature yellowing in leaves, suggesting that *SGRs* and *NYC* are the critical genes for accelerating chlorophyll degradation (Yamatani et al., 2013; Sakuraba et al., 2014). Here, the expression levels of *BrNYC1* and *BrSGRs* (*BrSGR1*, *BrSGR2*, and *BrSGRL*) were significantly suppressed in the H_2_O_2_ treatment group in comparison to that in the control group (Supplementary Figure S.3). Furthermore, accumulation of NYC1 and SGRs proteins in the H_2_O_2_ treatment group showed the same trend (Figure 3E). These results indicate that preharvest treatment with H_2_O_2_ maintained higher level of chlorophyll is correlated with the regulation on PAO/phyllobilin pathway.

Nevertheless, changes of chlorophyll content in senescent leaves is determined by the relative rates of chlorophyll degradation and synthesis processes that occur in chloroplasts (Hörtensteiner, 2006). Chlorophyll synthesis is still one of the critical processes for maintaining chlorophyll content, because chlorophyll synthesis process is detectable until the final stage of the lifespan in leaves (Hörtensteiner, 2006). The chlorophyll synthesis process in higher plants occurs through a specific pathway, in which Hems (HemA, L, B, and C), Chls (ChlE, M, I, and G), and CAO proteins are the key enzymes involved in the process (Figure 7B; Wang and Grimm, 2015). Our results showed that the main DEPs including HemA, ChlE, ChlM, ChlI, and CAO proteins were markedly upregulated in H_2_O_2_ treatment group in comparison with control group (Figure 3E), suggesting preharvest H_2_O_2_ treatment maintained leaf chlorophyll biosynthesis during storage. The results here indicate that preharvest treatment with exogenous H_2_O_2_ maintained chlorophyll content by simultaneously suppressing chlorophyll degradation and preserving chlorophyll synthesis in harvested cabbage. This can account for the higher chlorophyll *a* and *b* content in H_2_O_2_-treated Chinese flowering cabbage (Figures 4A,B).

Chlorophyll degradation during leaf senescence is a highly complex and ordered process can be triggered by internal age-dependent factors and external biotic and/or abiotic stresses (Zhang et al., 2021). After harvest, leafy vegetables are particularly prone to rapid dehydration due to their large surface area, and retention of water is crucial for their storage. It has been shown that excessive water loss can initiate chlorophyll degradation and induce rapid senescence in leaves, whereas an increase in proline can reduce water loss and delay leaf senescence (Jafari et al., 2019; Hanif et al., 2021). H_2_O_2_ acts as a signal molecule is involved in plant physiological metabolism, such as guard cell physiology, and helps plants against drought stress (Singh et al., 2017). It was showed that H_2_O_2_ plays an important role in ABA-induced stomatal closure and water retention (Watkins et al., 2017). The results here showed that water loss of H_2_O_2_ treatment group was lower than that of control group during storage (Figure 5A). The results were consistent with the regulations of proline content and leaf stomatal aperture in the H_2_O_2_ treatment group (Figures 5B–D). The biological process annotation also revealed that the main DEPs induced by H_2_O_2_ treatment were involved in regulation of stomatal opening and response to water deprivation (Figure 2D). These findings indicate that preharvest H_2_O_2_ treatment mediated leaf senescence during storage at least partly by reducing water loss.

**Figure 8:**
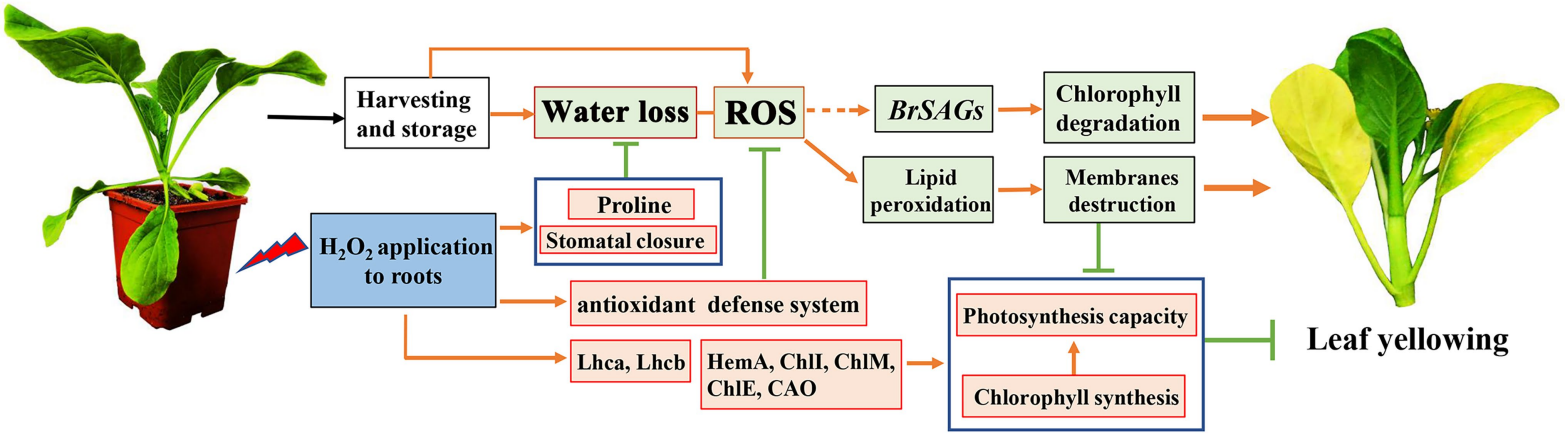
A proposed model of mechanism underlying preharvest H_2_O_2_ treatment delayed leaf yellowing of Chinese flowering cabbage during storage by alleviating oxidative stress and water loss. Red boxes indicate upregulation and green boxes indicate downregulation, being modulated by preharvest H_2_O_2_ treatment.

In addition to water loss, shading should be another major cause accelerating chlorophyll degradation and leaf yellowing when cabbages were stored under a weak light condition after harvest. It has been shown that weak light or darkness can initiate chlorophyll degradation and induce leaf senescence (Liebsch and Keech, 2016). Under dark environment, leaf senescence was manifested in an increase in ROS level followed by a subsequent transcriptional regulation of senescence marker genes *SAG12* (Rosenvasser et al., 2006). ROS is considered as the main factor to initiate the senescence program and chlorophyll degradation, and the expression of *Rbohs* were upregulated for production of endogenous ROS during the leaf aging process (Yu et al., 2019). ROS accumulation in cells can cause oxidative damage to membranes, lipids, proteins, and other macromolecules including DNA and RNA (Niu et al., 2020). This was confirmed in the present study that endogenous H_2_O_2_ in leaves gradually increased during storage and causes lipid peroxidation and membrane damage (Figures 6A–E). However, alleviation of water loss and oxidative stress are beneficial for maintaining membrane function and reduce chloroplast destruction (Figures 4–6). Undoubtedly, a high content of H_2_O_2_ may cause oxidative damage, leading to chlorophyll degradation, organelle dysfunction and plant senescence (Zhang et al., 2015). However, studies have also demonstrated that H_2_O_2_ at a low concentration serves as a secondary messenger in signaling pathways (Rogers and Munné-Bosch, 2016). Exogenous H_2_O_2_ can act as an activator to stimulate the antioxidant defense system, helping to reduce oxidative damage and cell senescence (Chumyam et al., 2019). Our experiments demonstrated that leaf yellowing of Chinese flowering cabbage was accelerated by high concentration of H_2_O_2_ application, whereas it was delayed by low concentrations (<50 mM) of exogenous H_2_O_2_ application to roots (Supplementary Figure S.1). It was showed that preharvest treatment with 10 mM H_2_O_2_ markedly delays the leaf senescence during storage. The treatment suppressed the accumulation of endogenous H_2_O_2_, reduced lipid peroxidation and protected the cell integrity (Figures 6A–F). These results may be attributed to the attenuation of oxidative stress that result from the activating of antioxidant defense system by preharvest treatment with low concentration of H_2_O_2_. It can be accounted for by the upregulated expression of ROS scavenging-related genes (*BrDHAR*, *BrMDHAR*, *BrSOD*, *BrCAT*, and *BrAPX*) and the suppression of ROS biosynthesis-related genes (*BrRbohB*, *BrRbohC*, and *BrRbohD*; Figures 6G,H). The above results are consistent with the GO functional annotation in proteomic analysis, which showed that the main DEPs induced by H_2_O_2_ treatment were involved in catalytic activity, antioxidant activity, and transmembrane transporter activity (Figure 2; Supplementary Figure S.2). The activation of antioxidant defense system in leaf tissues can be beneficial for reducing membrane lipid oxidation and suppressing the up-regulation of *SAG12* expression and finally delaying leaf senescence during storage (Figure 8).

Chloroplast is one of the most extensive membrane systems found in nature, and chloroplast membranes are rich in lipids and are susceptible to oxidative stress (Cook et al., 2021). Excessive accumulation of ROS is a major cause of chloroplast membranes destruction. The oxidative damage in thylakoid membranes will affect the photosynthetic capacity and accelerate senescence-associated degradation processes in leaves (Krieger-Liszkay et al., 2019). Maintaining strong antioxidant capacity should be a guarantee of the normal membranes function and photosynthetic activities (Exposito-Rodriguez et al., 2017). The decline of photosynthesis in leaves is correlated with the destruction of membranes and degradation of chlorophyll, as chlorophyll is the main component of thylakoid membrane–protein complexes such as PS II, PS I and the cytochrome *b6f* complex (Figures 7C,D; Sato et al., 2009). The proteins Lhcbs and Lhcas in PS II and PS I, combined with chlorophyll, function as antenna pigment coordinators that play an important role in light harvesting (Wei et al., 2016). During leaf senescence, the breakdown of light-harvesting chlorophyll protein complexes of LHCII is associated with the degradation of chlorophyll (Sakuraba et al., 2015). It was shown that chlorophyll *b* degradation catalyzed by CCEs in senescent leaves is required for the degradation of LHCII and thylakoid grana. In contrast, in the stay-green phenotype *NYC1* mutant, LHCII was selectively retained due to the chlorophyll *b* degradation significantly inhibited (Sato et al., 2009). The results here show that degradation of the light-harvesting complexes (Lhcb2, Lhcb4, Lhcb6, Lhca1, Lhca3, and Lhca4) in the control group is closely related to the decline of chlorophyll, which indicate abnormal chloroplast function and decline of photosynthetic capacity during leaf senescence. However, the Lhc proteins in leaves during storage were preserved by preharvest treatment with H_2_O_2_ (Figures 3C,D). These results suggest that protecting membranes from oxidative damage have crucial implications for the light-harvesting and photosynthetic capacity of leaves.

Moreover, the subunits in PS II and PS I, including PsbC, PsbE, PsbR, Psb27, and PsbG were also protected by preharvest H_2_O_2_ treatment (Figures 3C,D). These subunits are the core components of photosynthetic system. They embedded in membranes and assemble into photosynthetic complexes in plants (Figures 7C,D). Photosynthetic capacity in plants is closely related to some subunits in PS II, PS I, and ATP synthase (Suga and Shen, 2020). F-type ATPases are membrane integrated enzymes localized in the thylakoid membranes and act primarily as ATP synthases that generate the largest amount of ATP for photosynthesis activity (Kühlbrandt, 2019). In this study, preharvest 10 mM H_2_O_2_ treatment protected F-type ATPases (ATPF0B, ATPF1D; Figure 3C), implying more energy supply for leaf photosynthesis activity. The results of cellular components annotation in proteomic analysis revealed that the upregulated DEPs observed in the H_2_O_2_ treatment group are involved in components of chloroplast membranes (Figures 2F,G), and the KEGG pathways of light harvesting and photosynthesis (Figures 3A,B). This can account for the higher Fv/Fm value in H_2_O_2_ treatment group relative to the control group (Figures 4C,D), suggesting preharvest H_2_O_2_ treatment protect chloroplast function of Chinese flowering cabbage during storage. It was showed that the decline in photosynthesis-associated proteins and photosynthetic activity are the first events occurring at the onset of shade-induced leaf senescence (Brouwer et al., 2012; Krieger-Liszkay et al., 2019). Therefore, the protection in chloroplast function and photosynthesis activity in leaves can be beneficial for attenuating leaf senescence during storage under a weak light environment. These results were consistent with the expression level of senescence marker gene *BrSAG12* in leaves during storage (Figure 1D).

In conclusion, preharvest application of low H_2_O_2_ to roots reduced water loss by modulating proline and the stomatal aperture in Chinese flowering cabbage during storage. The treatment also activated the antioxidant defense system, reduced lipid peroxidation, protected cell membranes system, maintained cell integrity, and then suppressed the upregulation of senescence marker gene *BrSAG12*. Therefore, the treatment suppressed chlorophyll degradation, preserved chlorophyll content, protected the chloroplast function, and finally delayed the leaf yellowing (Figure 8). These results suggest that preharvest H_2_O_2_ treatment delay leaf yellowing during storage by alleviating water loss and oxidative stress. The findings provide possibility for developing new techniques to extend the shelf life of Chinese flowering cabbage or other leafy vegetables.

## Data Availability Statement

The raw data supporting the conclusions of this article will be made available by the authors, without undue reservation.

## Author Contributions

GW and SZ contributed to conception and design of the study and wrote sections of the manuscript. MP and YW organized the database. MP and ZC performed the statistical analysis. GW wrote the first draft of the manuscript. All authors contributed to the article and approved the submitted version.

## Funding

This work was financially supported by the National Natural Science Foundation of China (31901751), Natural Science Foundation of Guangdong Province (2020A1515010024), and Colleges Innovation Project of Guangdong (2018KQNCX017).

## Conflict of Interest

The authors declare that the research was conducted in the absence of any commercial or financial relationships that could be construed as a potential conflict of interest.

## Publisher’s Note

All claims expressed in this article are solely those of the authors and do not necessarily represent those of their affiliated organizations, or those of the publisher, the editors and the reviewers. Any product that may be evaluated in this article, or claim that may be made by its manufacturer, is not guaranteed or endorsed by the publisher.
