# Supplementary figures and images for: Preharvest Hydrogen Peroxide Treatment Delays Leaf Senescence of Chinese Flowering Cabbage During Storage by Reducing Water Loss and Activating Antioxidant Defense System
Source: Front Plant Sci. 2022 Mar 31;13:856646. doi: 10.3389/fpls.2022.856646 (PMC9009452; doi:10.3389/fpls.2022.856646)

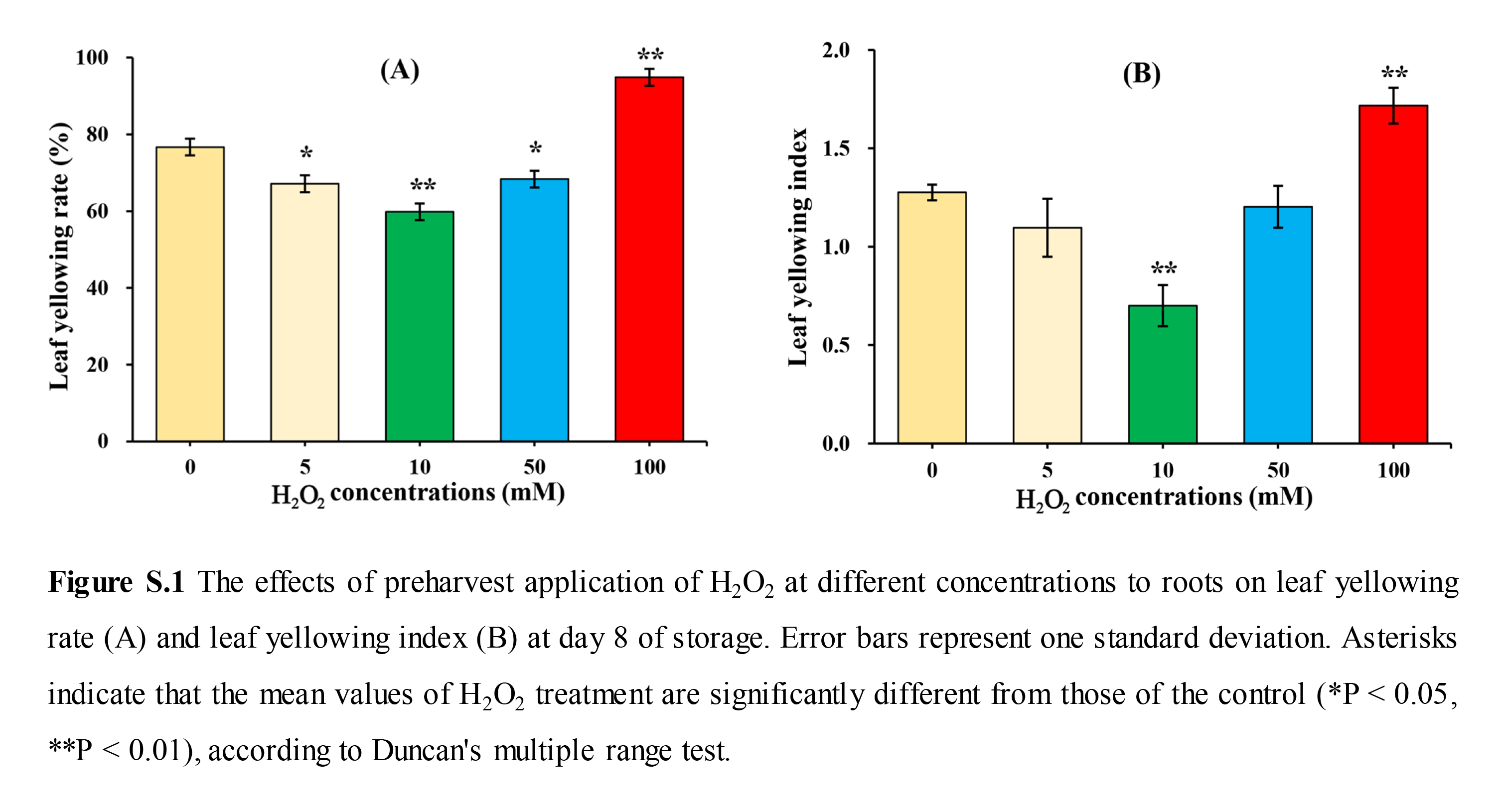

Supplement: Supplementary file 1 [file Image_1.TIF]

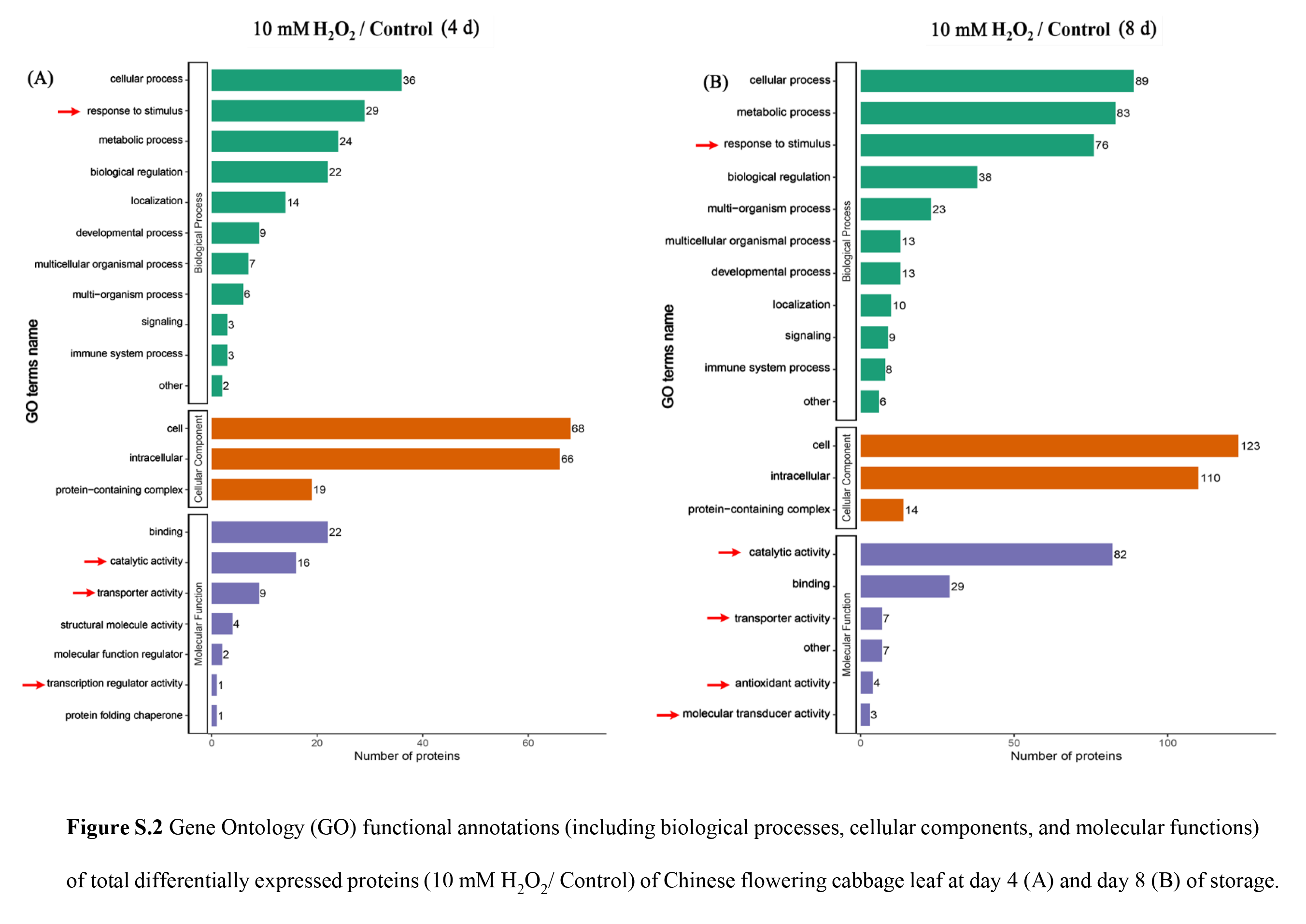

Supplement: Supplementary file 2 [file Image_2.TIF]

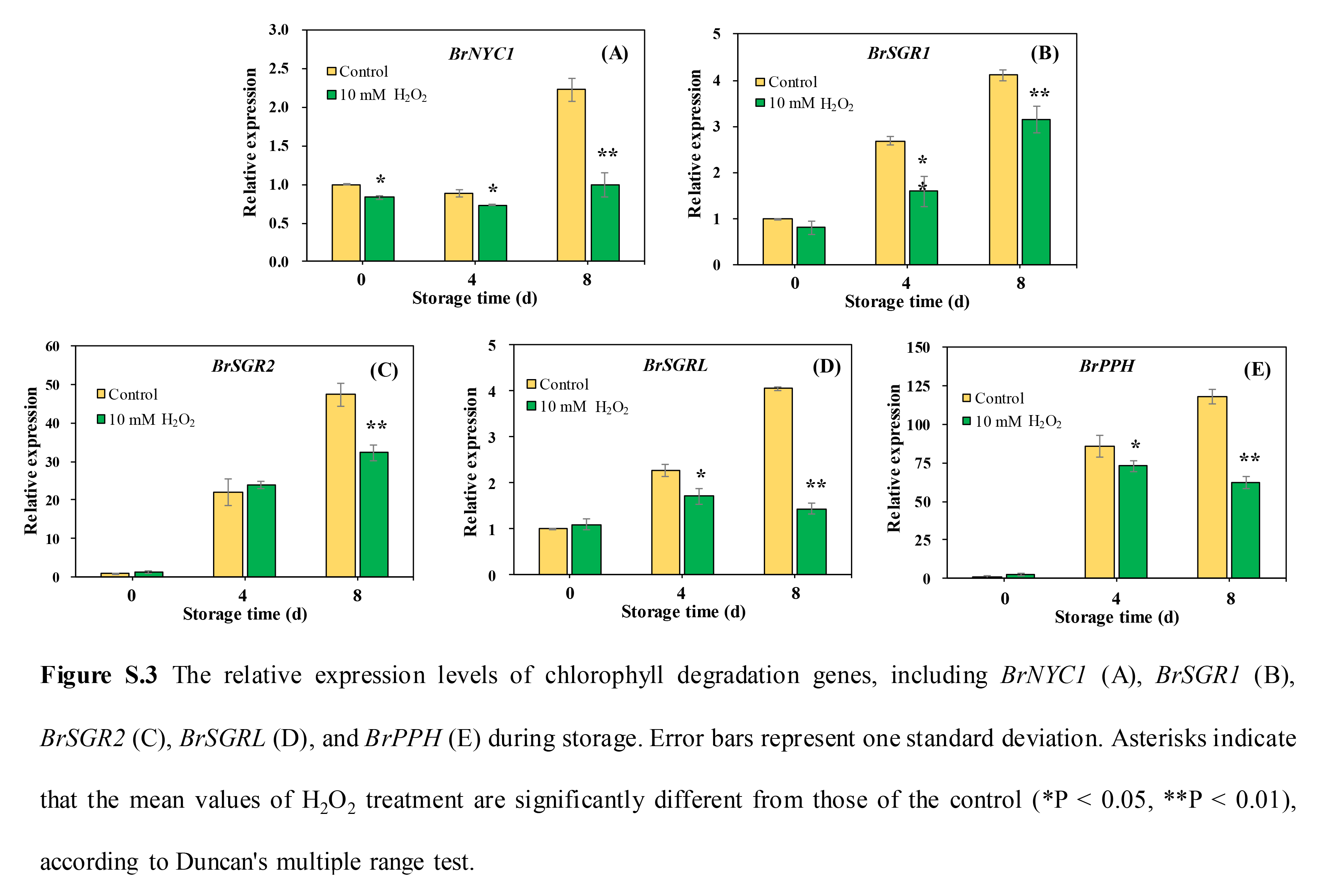

Supplement: Supplementary file 3 [file Image_3.TIF]
