# Supplementary material for: Preharvest Hydrogen Peroxide Treatment Delays Leaf Senescence of Chinese Flowering Cabbage During Storage by Reducing Water Loss and Activating Antioxidant Defense System
Source: Front Plant Sci. 2022 Mar 31;13:856646. doi: 10.3389/fpls.2022.856646 (PMC9009452; doi:10.3389/fpls.2022.856646)
Supplement: Supplementary file 4 [file Table_1.DOCX]

**Table 1.** Primers for Quantitative real-time PCR (qRT-PCR)

| Gene | Forward primer | Reverse primer |
| --- | --- | --- |
| *BrActin* | 5′-GGAGCTGAGAGATTCCGTTG-3′ | 5′-GAACCACCACTGAGGACGAT-3′ |
| *BrRbohB* | 5′-CCTCCCGATGTGAAGCCACT-3′ | 5′-AGCTTCCCCGAAACAACCGA-3′ |
| *BrRbohC* | 5′- GGATGGTCTGCCGTGGAGAA-3′ | 5′-CGTCTCGCCAACGCATCAAA-3′ |
| *BrRbohD* | 5′- TCCGACAACGAAAGCAACGC -3′ | 5′- TGAGTTCCTCCTCCGGGACT-3′ |
| *BrDHAR* | 5′-GCCAACGGGTTCTTCTGACC-3′ | 5′-ACGTCTGAATCAGCCACCCA-3′ |
| *BrMDHAR* | 5′-AGGCTGTGGTTGTTGGTGGA-3′ | 5′-TGTCGGCGGTGAAAAGCCTA-3′ |
| *BrSOD* | 5′-GCACCCGAGGATGCTAATCG-3′ | 5′-TACCACAAGCAACACGGCCT-3′ |
| *BrCAT* | 5′-GTTTGATCCTGTCCGGTGCG-3′ | 5′-CTCACGTTCAGACGGCTTGC-3′ |
| *BrAPX* | 5′-CAGTCATGGTGCCAACAGCG-3′ | 5′-GGAAGTCGTCCTTCGTCCGT-3′ |
| *BrNYC1* | 5′-CGTTGAGAGGCTGTCCAGTT-3′ | 5′-TGAAATCAAGCAGC-GGCCTA-3′ |
| *BrSGR1* | 5'-GTCCGCTTTGGGAAGCTACT-3' | 5'-ATGAGACCACGGAATCGAGC-3' |
| *BrSGR2* | 5'-AGTGGCCGAGTGGAAGAAAG-3' | 5'-AAGTTCCCGTCCCCATGAAC-3' |
| *BrSGRL* | 5'-ATGTGGCTGCAGAGCTTAGG-3' | 5'-CCTTGAGAGGTCCCCAACAC-3' |
| *BrPPH* | 5'-TATCTGATGCGCGGGTGGAT-3' | 5'-TTCCCGACCAATGCTGGACT-3' |
| *BrSAG12* | 5'-CACTGGCGGCTTAACCACTGAA-3' | 5'-GAAGATTGGCTGTATCCTACG GC-3' |
